# Progress in Diseases of the Blood

**Published:** 1901-03-02

**Authors:** 


### PROGRESS IN DISEASES OF THE BLOOD.

(*Continued from page* 335.)

**Pernicious Anæmia.**—A case of pernicious anæmia[14] of A. E. Barker's occurred in a man who had suffered from traumatic stricture of the intestines for seven years with ulceration of the intestines and chronic gastric catarrh. The anæmia only seems to have come on nine months before his death, and W. Hunter regards the case as illustrating his theory that this disease is due to an infective toxæmia chiefly streptococcal, originating in carious teeth, and especially apt to occur when intestinal lesions are present. The patient's teeth were unusually decayed, and ethmoidal suppuration was present, while the mucosa of the stomach and intestines, and the lymphatics showed streptococci and other organisms. C. L. Dana[15] thinks that a hæmolytic toxin is not sufficient unless there be a hereditary feebleness in the blood-forming organs. If such a poison was the sole cause there would be more marked general symptoms. Thus it is in the congenitably weak that syphilis and lead cause spinal scleroses, while in the strong these poisons may produce no such complications.

Besides arsenic and bone marrow Hommel's hæmatogen has been found useful in some cases, and is recommended by H. Meggit[16]. It is made from healthy blood, and contains hæmoglobin, salts, iron, and albumen in an easily absorbable form.

**Leukemia.**—Schwarz[17] reports a curious case which showed a number of peculiarly coloured myelocytes in the blood and also in the bone marrow. Müller thought such cells arose in the spleen, but their numbers in the marrow of this patient disprove that view. Osteosclerosis was also present here, which has been noticed in two other cases of leukemia, and has probably some connection with the production of myelocytes. In a case of lymphatic leukemia where ascites had occurred, W. Spencer[18] got rid of the ascites by establishing a collateral circulation. Laparotomy was performed, and a piece of the omentum was stitched to the abdominal wall. The ascites did not return while the patient was under observation, and her general health remained good. The operation has been employed of late in some cases of ascites due to cirrhosis of the liver. Percy Kidd[19] draws attention to the hæmorrhages in this disease. Sometimes purpuric spots, sometimes gastric and cerebral hæmorrhages, and occasionally others in the subcutaneous tissues forming large blood tumours, may be found. Uric acid gravel and calculi, peritonitis, either general or in the neighbourhood of the spleen, are now and then noted. In children, doubt may arise between leukemia, scurvy-rickets, and Hodgkin's disease. If lymphatic leucocytosis with myelocytes and nucleated red cells and some splenic enlargement is found, there is much to be said in favour of leukemia. Still there seems to be normally a relative deficiency of polynuclear cells in healthy children, and the blood in Hodgkin's disease may, towards the last, approach the leukemic type. Finally the blood may vary at different stages of leukemia, becoming at times nearly normal, so that counts should be made at intervals.

**Splenic Anæmia.**—R. Barclay Ness[20] reported an instance in a girl of 16. There was marked pallor, a huge spleen, slightly enlarged lymphatic glands, no hæmorrhages, and the temperature remained practically normal. The red cells were at one time as low as 2,300,000 with poikilocytosis, and a great reduction of hæmoglobin, the white numbering 10,000 in normal proportions, but with a few large mononuclear ones present. There was some evidence that the disease had existed for seven or more years. The case differed from the two types described by West, the first of which tends to a fatal termination after a short period, and frequently shows pyrexia and hæmorrhages, while the latter is found in children and ends in recovery for 40 per cent. of the cases. Here, too, hæmorrhages as well as gastric disturbances are frequent. Stockman has observed similar cases to that described by Ness, and regards the anæmia as due to hæmorrhages. W. K. Hunter thinks the disease is akin to splenic leukemia, just as he would compare Hodgkin's disease with lymphatic leukemia, the difference in each case being the absence of the blood changes seen in the respective types of leukemia. He, too, regards the anæmia as a result of blood destruction rather than of deficient formation. An exhaustive paper by Bovaird[21] describes a class of cases possibly belonging like these to the splenic anæmias. They seem to stand midway between the adult and infantile varieties. His own two cases were in children, and in one an autopsy showed endothelial hyperplasia of the spleen, together with similar changes in the liver and lymph glands. He gives reasons for denying that the growth was an epithelioma, and refers to other cases described by Gaucher, Weichselbaum, Collier, and Raymond in support of his view. Besides this affection of the spleen, simple anæmia and some hæmorrhages were alone observed. In the blood counts, while the red cells were diminished, the only peculiarity in the white cells was some excess of the large hyaline ones. The malady is steadily progressive, but may continue for many years—as many as twenty-five in Gaucher's patient. It is named by the author Primary Splenomegaly, and by Italian writers *Malattia del Banti*. Considerable discussion has arisen as to whether the disease is or is not splenic anæmia. Cominotti[22] shows that neither simple nor progressive anæmia may be present with hypertrophy of the spleen, for in a patient with a very large and painful spleen and recurrent epistaxis for ten years he found the red cells numbered seven millions and upwards, while in three recorded cases of tuberculosis of the spleen a similar state of things existed. He suggests that when the functional activity of this spleen is lessened fewer red cells are destroyed, and that the condition may not be uncommon after splenectomy. One such increase of red cells he found in a patient whose spleen had been removed seven years previously for malaria. Aloysius Kelly[23] discusses a somewhat acute form of splenic anæmia, an instance of which occurred in a girl of 22 with vaginal hæmorrhage, dyspnœa, diarrhœa, and high fever. He thinks that splenectomy, as advocated by Banti, should be avoided altogether in pyrexial and acute cases.

**Chlorosis.**—Seymour Taylour[24] finds that this affection is present in 8 per cent. of his hospital outpatients and that if it continues long enough without treatment gastric ulceration is sure to follow. He regards it as due to defective assimilation. In the dyspeptic type he treats the stomach condition and constipation thoroughly before giving iron. In the neurotic form he adds valerian to the digestive mixtures which precede ferruginous treatment. In the cases where extreme pallor and dyspnœa exist, iron with arsenic is given at an early stage, and in all the hæmoglobin should be tested every week, and if no progress is being made the preparation of iron should be changed. Fresh carbonate, albuminate of iron, and bipalatinoids are perhaps the most useful forms of iron. Senator[25] recommends the water of alkaline, and later on of ferruginous springs, organic salts, and if gastric catarrh is present the ammonio-chloride of iron. Among new methods the sweat cure by hot-air and other baths is useful either by itself or as an adjuvant to other measures. Hemmeter[26] sums up the causes of anæmia as deficient food or deficient iron-bearing compounds in food, such as nucleo-albumens, excess of sulphur compounds in the digestive tract which destroy the iron of the food, or lastly, in septic conditions, failure of metabolism and of absorption of the food. He thinks that the inorganic preparations of iron are satisfactory, and shows that iron is absorbed even when there is no HCl. in the gastric juice, contrary to Porter's theory. He relates the case of a patient who had no gastric juice at all from atrophy of the stomach. He was treated with carbonate of iron, and yet after death iron was demonstrated in the epithelium of the duodenum. The dyspnœa of anæmia, according to J. Henton White,[27] is associated with dilation of the pulmonary artery and conus arteriosus of the right ventricle. Thus dulness may extend to the third left cartilage, or even to the clavicle, the apex beat being often raised to the fourth space close to the nipple line. The pulmonary second sound is accentuated and the left ventricle is but little affected. Moreover there is some paresis of the vasomotor system, so that the size of the radial artery is decreased on standing up and enlarges on lying down, except in cases where high tension exists. Probably the same occurs in the pulmonary system, and hence on exertion the extra blood has to be driven through the lungs without the aid of dilatation of the vessels, which results in the pressure shown by the accentuated second sound and the dyspnœa. Nathan[28] holds that mineral iron is converted into organic compounds in the intestine before it is absorbed, hence he advises the use of albuminates and iron somatose in preference, and shows by microscopical examination of the intestines that such compounds are readily absorbed in large quantities. Lereboullet[29] has given cacodylate of iron by the mouth, 5 to 30 centigrammes daily, and hypodermically, 3 to 10 daily, and found no local or general disturbance. It counteracts both the fall in hæmoglobin and in the number of the red corpuscles. In tuberculous anæmia it is peculiarly valuable, and albuminuria in such patients frequently improves after its use. Tallgrist[30] produced in dogs severe anæmias by giving certain blood-destroying drugs, and concludes that the body during periods of hæmolysis retains the iron as a reserve for the building up of hæmoglobin and other compounds during recovery. In acute attacks he finds that recovery is slower than after chronic ones, which he believes to be due to the smaller accumulated reserve of iron. These theories may explain some facts in pernicious anæmia and other similar diseases.

[14] Lancet, July 21st. [15] Med Rec.. Dec. 1st. [16] Lancet, Aug. 4th. [17] Med. Press, Nov. 7th. [18] Lancet, June 16th. [19] Clin. Jour., July 11th. [20] Glasgow Med. Jour., December. [21] Amer. Jour. Med. Sc., October, [22] Med. Chron., November. [23] Med. News, July 12. [24] Clin. Jour., Jan. 2. [25] Merck's Archives, October. [26] Med. Chron., October. [27] Birmingham Med. Rev., October. [28] Therapist., Sept. 15th. [29] Brit. Med. Jour., Oct. 13. [30] Brit. Med. Jour., Jan. 19.

